# *Tremella fuciformis* Extract Evokes Similar Effect as Hyaluronic Acid on Wound Healing but Through Different Mechanisms in Human Dermal Fibroblasts

**DOI:** 10.3390/molecules31132354

**Published:** 2026-07-03

**Authors:** Katarzyna Wolosik, Gabriela Gasiewska, Dorota Wrzesniok, Jerzy Palka, Arkadiusz Surazynski

**Affiliations:** 1 Independent Cosmetology Laboratory, Medical University of Bialystok, Kilinskiego 1, 15-089 Bialystok, Poland; 2 Department of Medicinal Chemistry, Medical University of Bialystok, Kilinskiego 1, 15-089 Bialystok, Poland; gabriela.gasiewska@sd.umb.edu.pl (G.G.); jerzy.palka@umb.edu.pl (J.P.); arkadiusz.surazynski@umb.edu.pl (A.S.); 3 Department of Pharmaceutical Chemistry, Faculty of Pharmaceutical Sciences in Sosnowiec, Medical University of Silesia in Katowice, Jagiellonska 4, 41-200 Sosnowiec, Poland; dwrzesniok@sum.edu.pl

**Keywords:** *Tremella fuciformis*, human dermal fibroblasts, hyaluronic acid, collagen biosynthesis, prolidase activity, wound healing, scratch assay, dermocosmetics, skin regeneration

## Abstract

*Tremella fuciformis* extract (TFE) is used in dermocosmetic formulations due to its moisturising, antioxidant, and skin-supportive properties. The present study compared the effects of commercial TFE and hyaluronic acid (HA) on selected functions of human dermal fibroblasts (HDF). The cells were treated with TFE at concentrations of either 200 µg/mL or 500 µg/mL, or with HA at a concentration of 500 µg/mL. The following parameters were the focus of the study: cell viability, DNA and collagen biosynthesis, prolidase activity, scratch-wound closure, and immunofluorescence of selected signalling- and extracellular matrix-related markers. The findings of this study demonstrate that neither TFE nor HA had any effect on HDF viability. TFE led to a significant increase in DNA biosynthesis at both concentrations, while HA had no significant effect. The synthesis of collagen was found to be considerably elevated by both HA and TFE500, with no such effect observed in the presence of TFE200. Prolidase activity was observed to be highest in the HA group and also elevated in the TFE500 group; however, these results should be regarded as descriptive due to the nature of the pooled-sample data. Immunofluorescence analysis revealed increased phosphorylated protein kinase B (p-AKT) fluorescence in images treated with TFE, while phosphorylated mechanistic target of rapamycin (p-mTOR) remained close to the control level. Higher levels of β1-integrin, Insulin-Like Growth Factor-I Receptor (IGF-1R), prolidase, and phosphorylated Extracellular Signal-Regulated Kinases (p-ERK1/2) fluorescence were also observed in selected groups. The mean scratch-wound closure was found to be highest for TFE500. Overall, TFE was found to be associated with DNA biosynthesis, whereas HA and TFE500 were found to enhance collagen biosynthesis. Further studies are required to confirm biological reproducibility and the mechanism.

## 1. Introduction

The skin is a dynamic and multifunctional organ that serves not only as a physical barrier protecting the body against environmental stressors but also as an active regulator of physiological homeostasis. It is continuously exposed to external factors such as ultraviolet radiation (UV), oxidative stress, or mechanical damage, which can impair its structural and functional integrity [1,2]. Within the dermal layer, human dermal fibroblasts (HDF) play a crucial role in maintaining skin architecture by regulating extracellular matrix (ECM) synthesis, remodelling, and repair processes. Alterations in fibroblast activity are closely associated with skin ageing, reduced regenerative capacity, and impaired wound healing [3,4].

The ECM of the dermis is composed primarily of collagen, which provides tensile strength, elasticity, and structural support [3,5]. Collagen homeostasis is a controlled process involving a balance between synthesis and degradation. One of the key enzymes involved in this process is prolidase, which catalyses the final step of collagen degradation by releasing proline from imidodipeptides containing C-terminal proline or hydroxyproline. This mechanism enables the recycling of proline for collagen resynthesis, thereby linking collagen turnover with ongoing matrix remodelling. Consequently, both collagen biosynthesis and prolidase activity are widely recognised as important indicators of fibroblast metabolic activity and ECM dynamics [5].

Dermal fibroblasts also play a significant role in wound healing, a complex and multistage process involving coordinated cell migration, proliferation, and matrix remodelling. In the early stages of wound repair, fibroblast migration into the damaged area is essential for restoring tissue continuity, followed by increased synthesis of ECM components. The efficiency of these processes directly influences the rate and quality of tissue regeneration. Therefore, compounds capable of modulating fibroblast function without inducing excessive proliferation are of particular interest in both biomedical and dermocosmetic research [6].

Hyaluronic acid (HA) is an endogenous glycosaminoglycan that plays an important role in skin hydration, ECM organisation, and tissue repair [7,8,9]. The biological effects of HA are determined by a number of factors, including its molecular weight, concentration, exposure time, formulation, and cellular context [8,9,10]. Due to the fact that HA has been extensively researched and is commonly used in dermatological and dermocosmetic products, it can be considered a valuable reference point for the evaluation of natural skin-supportive ingredients that have not been fully standardised [7,8].

*Tremella fuciformis*, commonly known as snow mushroom or silver ear mushroom, is an edible basidiomycete that forms a soft, gelatinous fruiting body with a high water-binding capacity [11,12,13]. The fungus has attracted increasing attention in the fields of food, pharmaceutical, and cosmetic research because its fruiting body contains abundant water-soluble, high-molecular-weight polysaccharides [11,14]. The incorporation of extracts obtained from *Tremella fuciformis* into topical formulations has increased, with these extracts being used as moisturising and skin-conditioning ingredients [13,15].

The major bioactive components described in *Tremella fuciformis* are structurally heterogeneous acidic heteropolysaccharides, commonly referred to as *Tremella fuciformis* polysaccharides (TFP) [11,12]. These polysaccharides are characterised by a mannose-rich backbone and branched side chains composed of monosaccharides, including xylose, glucuronic acid, fucose, galactose, glucose, arabinose, and rhamnose [12,16]. The uronic acid content of these substances contributes to their polyanionic character and strong affinity for water [11,14]. However, it should be noted that molecular weight, branching, acetylation, monosaccharide composition, and biological activity can vary according to fungal strain, cultivation conditions, extraction procedure, and degree of purification [11,12,14,16]. Consequently, it is not possible to assume that commercial *Tremella fuciformis*-derived preparations have equivalent chemical compositions or biological activities.

The most well-established dermocosmetic property of TFP-containing formulations is their capacity to bind water and form a hydrophilic film. This process may support skin hydration and reduce transepidermal water loss (TEWL) [11,13]. Experimental evidence also indicates antioxidant, cytoprotective, immunomodulatory, and anti-inflammatory activities [11,12,13,16,17,18]. Isolated TFP protected human skin fibroblasts against hydrogen peroxide-induced injury in association with SIRT1-related responses [17]. TFP also reduced UVA-induced damage in HDFs through responses involving the Nrf2/Keap1 antioxidant pathway [18]. Furthermore, a *Tremella fuciformis*-based formulation has been demonstrated to promote the closure of scratches in cultured human fibroblasts and keratinocytes [19]. In an animal model of atopic dermatitis, TFP administration has been shown to modulate inflammatory and immune responses [20]. Most of the available evidence concerning TFP is, nevertheless, derived from *in vitro* or animal studies [13,17,18,19]. Furthermore, studies frequently examine isolated or specifically characterised polysaccharide fractions rather than commercial extracts. The biological effects reported for purified TFP should therefore not be automatically assigned to every commercial *Tremella fuciformis* extract (TFE).

Macrofungal extracts are increasingly investigated as sources of polysaccharides, phenolic compounds, sterols, proteins, glycosides, and triterpenes with potential antioxidant, photoprotective, anti-inflammatory, moisturising, and ECM-supportive properties [15]. Within this extensive group, *Tremella fuciformis*-derived preparations are of particular scientific interest because they combine pronounced hydrophilicity and film-forming properties with reported cytoprotective and skin-cell-modulating effects [13,15,17,18,19]. This distinguishes TFE from natural extracts, which are investigated predominantly for low-molecular-weight antioxidant or anti-inflammatory constituents.

TFE and HA, therefore, have some functional properties in common but should not be considered molecularly analogous. HA is a relatively well-defined linear glycosaminoglycan for which there is a substantial evidence base in dermatology and cosmetology [7,8,10]. In contrast, TFE is a compositionally heterogeneous commercial material whose reported activities are supported predominantly by in vitro and animal studies [11,13,15]. In view of these findings, TFE may be considered a potentially complementary dermocosmetic ingredient rather than a direct substitute for HA. A direct comparison may reveal whether the two materials preferentially affect different fibroblast functions, including proliferation, collagen metabolism, and scratch-wound closure.

The present study used a commercial TFE, not an isolated or chemically defined TFP fraction. The preparation was described by the manufacturer as polysaccharide-rich, but no independent fractionation or chemical characterisation has been performed. Consequently, the observed biological effects are attributed to TFE as a whole and cannot be assigned exclusively to TFP.

As demonstrated in the relevant literature, the proteins β1-integrin and Insulin-Like Growth Factor-I Receptor (IGF-1R), in conjunction with phosphorylated protein kinase B/mechanistic target of rapamycin (AKT/mTOR)- and Extracellular Signal-Regulated Kinases (ERK1/2)-related signalling pathways, have been shown to play a pivotal role in the proliferation, migration, survival, and collagen-related responses of fibroblasts [21,22,23,24]. The evaluation of their immunofluorescence may provide supportive information about cellular response patterns. However, it is important to note that changes in fluorescence intensity alone do not provide sufficient evidence to establish pathway activation or causal molecular mechanisms.

Despite the growing interest in *Tremella fuciformis*-derived dermocosmetic ingredients, direct comparative evidence concerning the effects of commercial TFE and HA on HDF viability, DNA biosynthesis, collagen metabolism, prolidase activity, signalling-related immunofluorescence, and scratch-wound closure remains limited. The present study, therefore, compared TFE and HA as functionally overlapping but non-equivalent materials. The aim of this study was to determine whether TFE produces fibroblast responses similar to those induced by HA, or whether it exhibits a distinct profile that could support its use as a complementary dermocosmetic ingredient.

## 2. Results

### 2.1. The Effect of TFE and HA on Cell Viability, DNA Biosynthesis, and p-Akt/mTOR Immunofluorescence in HDF

**Figure 1:**
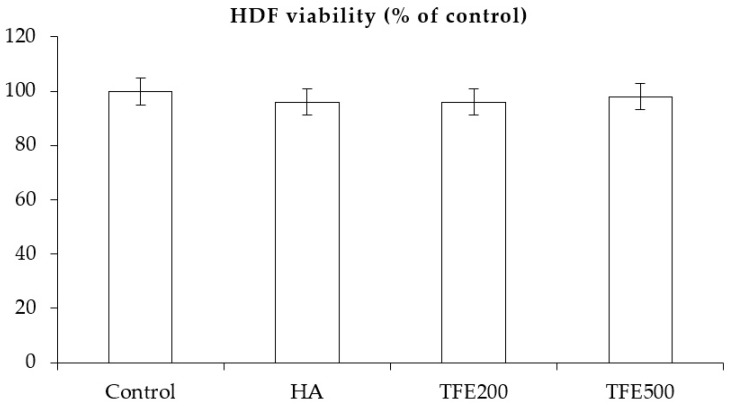
Viability of human dermal fibroblasts (HDF) treated with *Tremella fuciformis* extract (TFE) and hyaluronic acid (HA)**.** Cells were treated with HA at 500 µg/mL or TFE at 200 and 500 µg/mL. Cell viability was determined using the MTT assay and expressed as a percentage of the control. Five technical replicates (five wells) were analyzed per condition within the same experiment. Data are presented as mean ± SD.

The effect of TFE and HA on HDF viability was evaluated using the MTT assay. As demonstrated in Figure 1, the treatment of cells with HA (500 µg/mL) or TFE (200 µg/mL, 500 µg/mL) resulted in the maintenance of HDF viability. No differences in cell viability were observed between the control and treated cells (Table S1). The findings of this study suggest that both HA and the TFE concentration used in this experiment did not have a significant impact on the viability of HDF cells within the experimental parameters.

**Figure 2:**
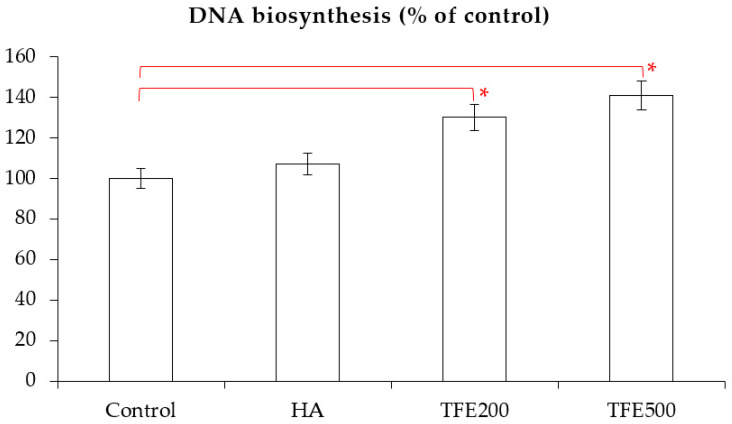
Effects of TFE and HA on DNA biosynthesis in HDF. Cells were treated with HA at 500 μg/mL or TFE at 200 and 500 μg/mL. DNA biosynthesis was expressed as a percentage of the untreated control. Three independent experiments were performed, with three technical replicates per condition in each experiment. Data are presented as mean ± SD (n = 3 independent experiments). Statistical significance was assessed using one-way ANOVA followed by Dunnett’s multiple-comparisons test; *p* < 0.05 was considered significant (*).

The effect of the tested preparations on cell proliferation was evaluated by measuring DNA biosynthesis. A one-way analysis of variance (ANOVA) was performed, which demonstrated a statistically significant overall difference among the experimental groups. Dunnett’s multiple-comparisons test demonstrated that HA at 500 µg/mL exerted no significant influence on DNA biosynthesis in comparison with the control, whereas TFE at 200 and 500 µg/mL caused a significant increase in this parameter. The findings suggest that TFE may promote a proliferative response in HDFs under the applied experimental conditions (Table 1, Figure 2).

**Figure 3:**
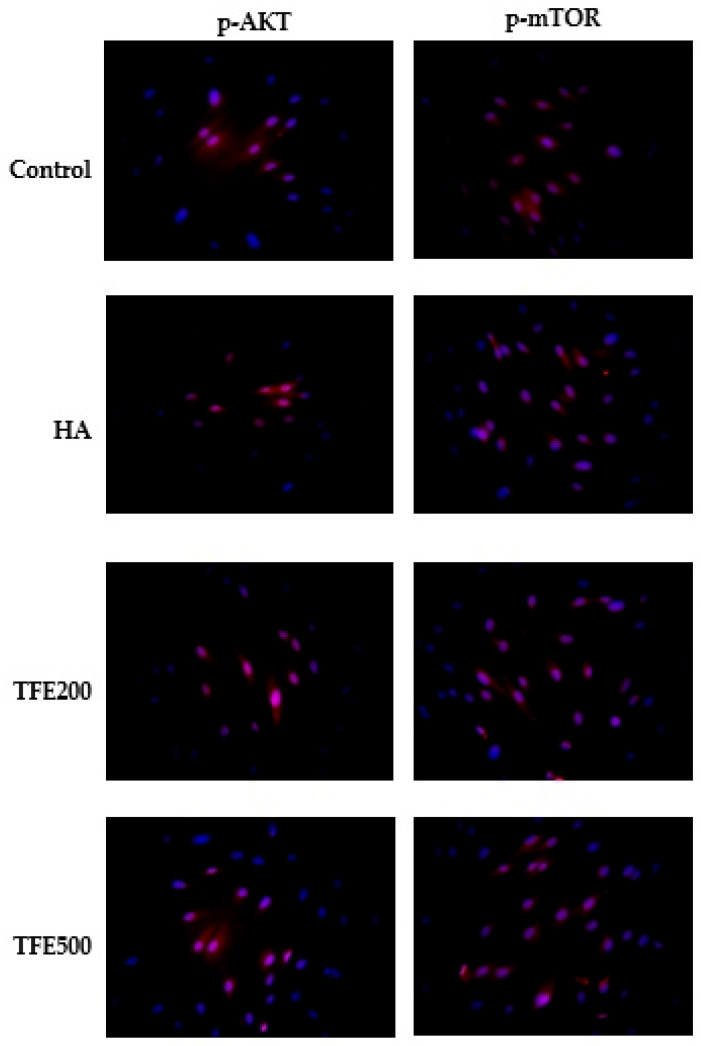
Immunofluorescence images of phosphorylated protein kinase B (p-AKT) and phosphorylated mechanistic target of rapamycin (p-mTOR) in HDF treated with HA and TFE. Cells were treated with HA at 500 μg/mL or TFE at 200 and 500 μg/mL. The blue fluorescence channel shows cell nuclei, whereas the red fluorescence channel represents p-AKT or p-mTOR fluorescence. Images were acquired using a 20× objective. One microscopic field per condition was analyzed. Quantitative analysis of the corresponding fluorescence images is presented in Table 2.

The effects of TFE and HA on p-AKT and p-mTOR immunofluorescence are presented in Figure 3. Descriptive quantitative image analysis was performed by the following steps: first, the integrated fluorescence was corrected for background intensity; second, the result was normalised to the number of Hoechst-positive nuclei; and third, the values were expressed relative to the control, which was set to 100%. The results of quantitative analysis have been presented in Table 2, whereas the complete image-analysis measurements, including analysed area, integrated density, mean background fluorescence, nuclei count, and background-corrected integrated fluorescence, are provided in the Supplementary Materials (Table S2).

The p-AKT fluorescence intensity per Hoechst-positive nucleus was found to be increased in the HA, TFE200, and TFE500 images in comparison to the control image, with values of 112.9%, 154.2%, and 223.8% of the control value, respectively. In contrast, the p-mTOR fluorescence intensity was found to be lower in the HA image, with levels remaining close to the control level in the TFE200 and TFE500 images. The descriptive findings indicate higher p-AKT fluorescence in the TFE-treated images, whereas no comparable increase in p-mTOR fluorescence was observed. Because this analysis was based on one representative image per condition, these data should be interpreted as supportive observations rather than statistically validated evidence of pathway activation.

### 2.2. Collagen Biosynthesis, Prolidase Activity, and β1-Integrin and IGF-1R Immunofluorescence in HDF Treated with TFE and HA

**Figure 4:**
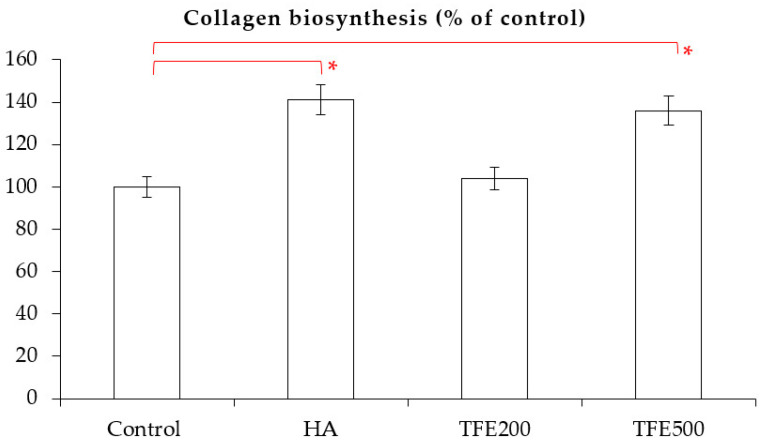
Effects of TFE and HA on collagen biosynthesis in HDF. Cells were treated with HA at 500 μg/mL or TFE at 200 and 500 μg/mL. Three technical replicates (three wells) were analysed per condition within a single experiment. Collagen biosynthesis was expressed relative to the untreated control, which was set to 100%. Data are presented as mean ± SD. Differences among groups were analysed using one-way ANOVA followed by Dunnett’s multiple-comparisons test, comparing each treatment group with the control. *p* < 0.05 were considered statistically significant (*).

The evaluation of collagen biosynthesis was conducted by measuring the incorporation of 5-[^3^H]-proline into collagenase-sensitive proteins. As demonstrated in Figure 4, collagen biosynthesis levels achieved 141.2% of the control value in the HA group, 104.0% in the TFE200 group, and 135.6% in the TFE500 group. The one-way analysis of variance (ANOVA) revealed a significant overall difference among the experimental groups (F(3.8) = 35.68, *p* < 0.001). Dunnett’s multiple-comparisons test demonstrated that HA and TFE500 significantly increased collagen biosynthesis in comparison with the control group. In contrast, TFE200 did not differ significantly from the control (Table 3). Therefore, an increased biosynthesis of collagen was observed following treatment with HA and the higher concentration of TFE.

**Figure 5:**
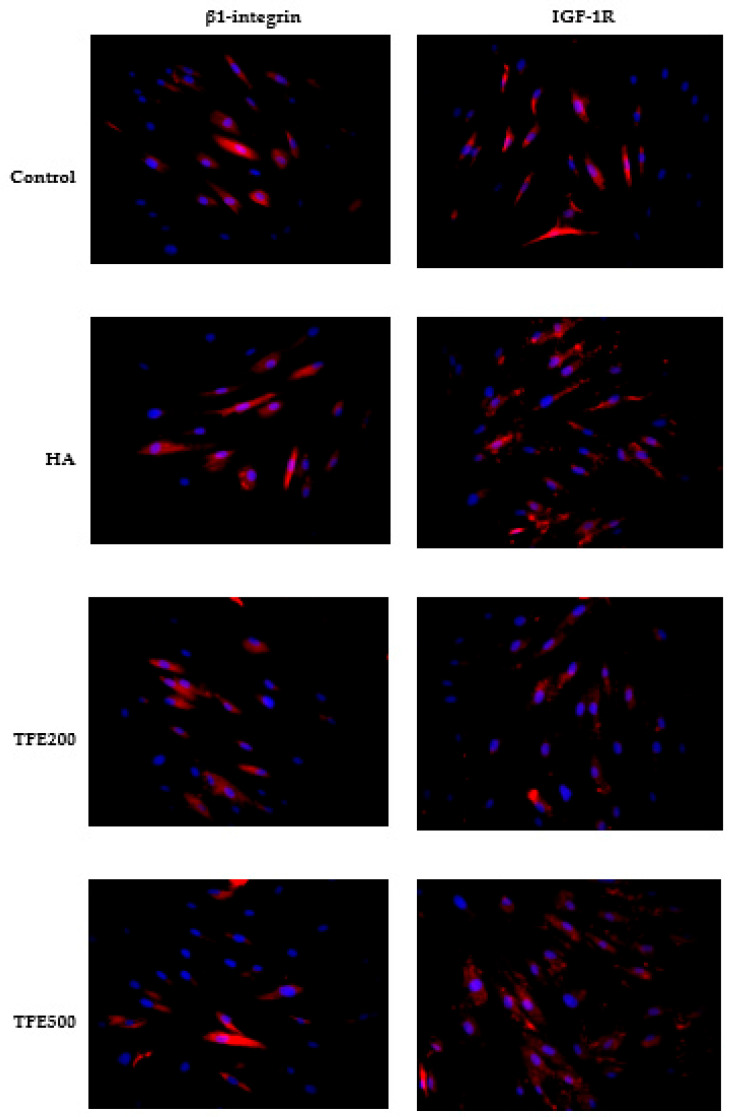
Immunofluorescence images of β1-integrin and Insulin-Like Growth Factor-I Receptor (IGF-1R) in HDF treated with HA and TFE. Cells were treated with HA at 500 μg/mL or TFE at 200 and 500 μg/mL. The blue fluorescence channel shows cell nuclei, whereas the red fluorescence channel represents β1-integrin or IGF-1R fluorescence. Images were acquired using a 20× objective. One microscopic field per condition was analyzed. Quantitative analysis of the corresponding fluorescence images is presented in Table 4.

Further characterisation of cellular responses associated with ECM regulation was conducted through the evaluation of β1-integrin and IGF-1R immunofluorescence (Figure 5). Quantitative descriptive analysis indicated that the fluorescence intensity of β1-integrin per Hoechst-positive nucleus was 141.96% of the control value in the HA group, 126.22% in the TFE200 group and 146.85% in the TFE500 group. IGF-1R fluorescence intensity was found to be 126.19%, 109.28% and 161.42% of the control value in the HA, TFE200 and TFE500 groups, respectively. The quantitative results normalised by area are presented in Table 4, while the complete image analysis measurements, including analysed area, integrated density, mean background fluorescence, nuclei count and background-corrected integrated fluorescence, are provided in the Supplementary Materials (Table S3). The images of HA and TFE500 showed higher levels of β1-integrin fluorescence compared to the control image, while the TFE500 image showed the highest levels of IGF-1R fluorescence. The higher fluorescence intensity of both markers in the TFE500 image was consistent with the increased collagen biosynthesis detected at this concentration. These findings should be considered descriptive because only one image per condition was analysed. Therefore, they do not provide statistical evidence and cannot establish a causal relationship between β1-integrin or IGF-1R fluorescence and collagen biosynthesis.

**Figure 6:**
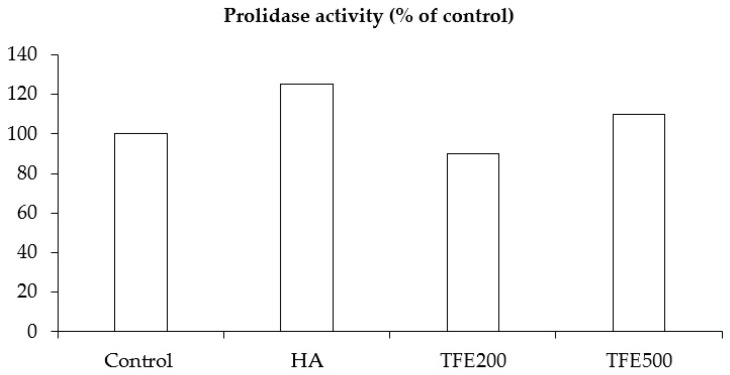
Prolidase activity in HDF treated with HA and TFE. Cells were treated with HA at 500 µg/mL or TFE at 200 and 500 µg/mL. Prolidase activity is expressed as a percentage of the control, which was set to 100%. The measurements were obtained from pooled samples; therefore, the data are descriptive and no statistical analysis was performed.

Prolidase activity was assessed. This was used as an additional indicator of collagen-related metabolic activity in HDF. The measurements were obtained from pooled samples. The data are presented descriptively. Accordingly, prolidase activity was used only as supportive information and was not treated as a statistically validated endpoint. This means they do not include statistical analysis. Compared to the control value of 100%, the prolidase activity was found to be 125% in the HA group, 90% in the TFE200 group and 110% in the TFE500 group (Figure 6).

**Figure 7:**
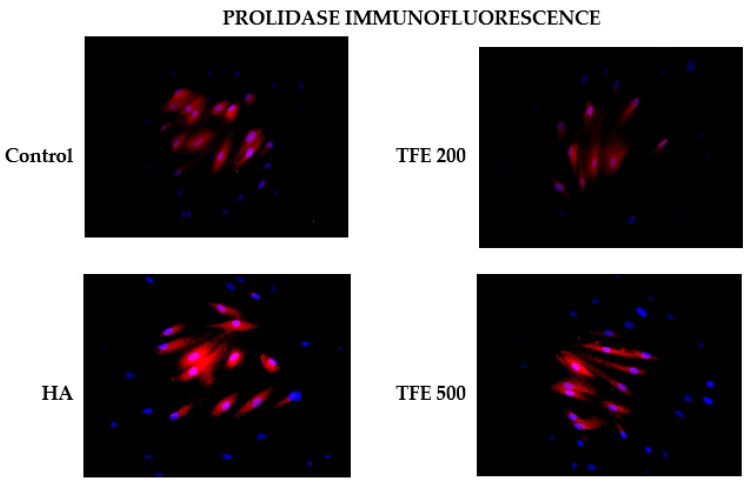
Prolidase immunofluorescence in HDF treated with HA and TFE. Cells were treated with HA at 500 μg/mL or TFE at 200 and 500 μg/mL. The blue fluorescence channel shows cell nuclei, whereas the red fluorescence channel represents prolidase fluorescence. Images were acquired using a 20× objective. One microscopic field per condition was analyzed. Quantitative analysis of the corresponding original fluorescence images is presented in Table 5. For presentation purposes, identical brightness, contrast, and sharpening adjustments were applied uniformly to all displayed images; quantitative analysis was performed using the original unprocessed images.

Prolidase immunofluorescence was also evaluated (Figure 7). Descriptive quantitative image analysis revealed that background-corrected prolidase fluorescence intensity per Hoechst-positive nucleus was 189.5% of the control value in the HA group, 76.7% in the TFE200 group and 156.0% in the TFE500 group. The HA image showed the highest prolidase fluorescence, whereas TFE500 was also found to be associated with higher fluorescence than the control. The normalised quantitative results are presented in Table 5, and the complete image analysis measurements, including analysed area, integrated density, mean background fluorescence, nuclei count and background-corrected integrated fluorescence, are provided in the Supplementary Materials (Table S4). These descriptive findings suggest an association between HA treatment, higher prolidase activity, and higher prolidase fluorescence, but do not statistically confirm or demonstrate that prolidase directly mediated the increase in collagen biosynthesis.

The immunofluorescence pattern was broadly consistent with the results of the prolidase activity analysis, particularly for HA and TFE500. The results suggest an association between HA treatment and increased prolidase activity and fluorescence associated with prolidase, but they do not confirm that prolidase directly increased collagen biosynthesis.

### 2.3. Effects of TFE and HA on Scratch-Wound Closure and p-ERK1/2 Immunofluorescence in HDF

**Figure 8:**
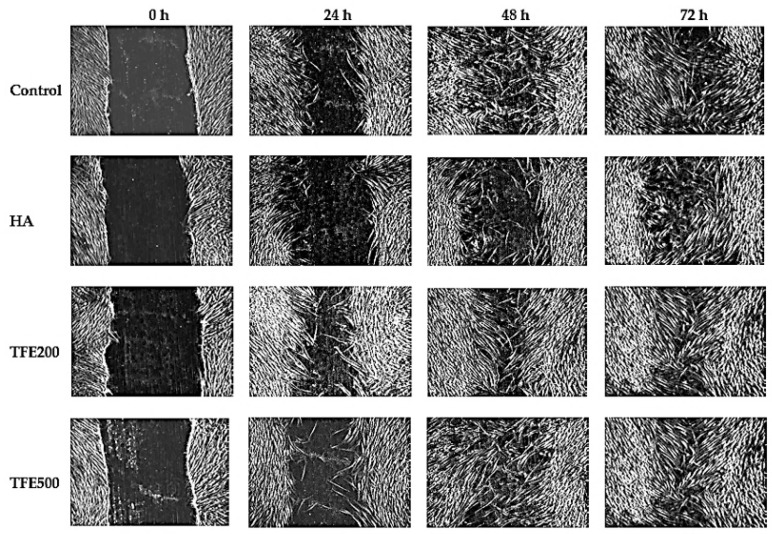
Time-dependent scratch-wound closure in HDF treated with HA and TFE. Representative microscopic images of untreated control cells and cells treated with HA at 500 μg/mL or TFE at 200 and 500 μg/mL are shown at 0, 24, 48, and 72 h after scratch formation. One representative image is presented for each condition and time point. Image were sharpened for presentation purposes only.

The effects of TFE and HA on scratch-wound closure in HDFs were evaluated using an in vitro scratch assay. Figure 8 presents representative microscopic images recorded at 0, 24, 48 and 72 h after scratch generation. Visual assessment showed progressive narrowing of the scratch area over time in all experimental groups. Compared with the untreated control group, scratch closure appeared more pronounced in the HA- and TFE-treated groups, particularly in cells treated with TFE500.

A quantitative analysis was performed for the 0–24 h interval using the Fiji/ImageJ software programme. This was the last interval for which the remaining scratch area could be consistently identified and defined in all experimental groups. Wound closure was calculated from the change in wound area relative to its initial value according to the equation described in Section 4.2.10 of the Materials and Methods.

**Figure 9:**
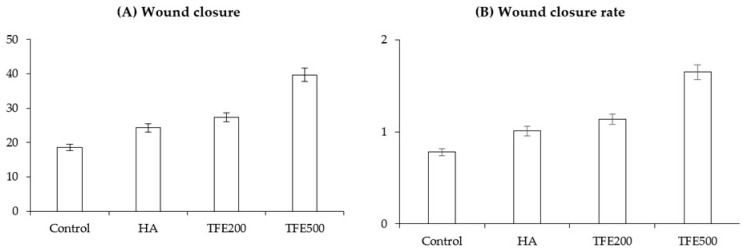
Quantitative analysis of scratch-wound closure in HDFs during the first 24 h after scratch formation. (**A**) Scratch-wound closure after 24 h, expressed as v%/24, and (**B**) wound-closure rate, expressed as %v/h. Cells were treated with HA at 500 µg/mL or TFE at 200 and 500 µg/mL. Data are presented as mean ± SD.

At 24 h, the mean percentage of wound closure was 18.66 ± 8.38% in the control group, 24.30 ± 4.43% in the HA group, 27.33 ± 11.57% in the TFE200 group and 39.70 ± 16.27% in the TFE500 group. While the TFE500 group exhibited the highest mean wound-closure value, the differences between the treatment groups and the control group were not statistically significant. Therefore, the scratch-wound closure data should be interpreted as indicating a trend under the applied in vitro conditions rather than a statistically confirmed treatment effect. The quantitative results are presented in Figure 9 and Table S5.

The scratch area was found to be extensively or nearly completely closed at 48 and 72 h. The remaining wound area was too small and irregular to be reliably detected and delineated using Fiji/ImageJ. Therefore, quantitative measurements at these later time points would have introduced substantial uncertainty. Consequently, the images taken at 48 and 72 h are presented as qualitative documentation of the later stages of scratch closure and were not included in the quantitative analysis.

**Figure 10:**
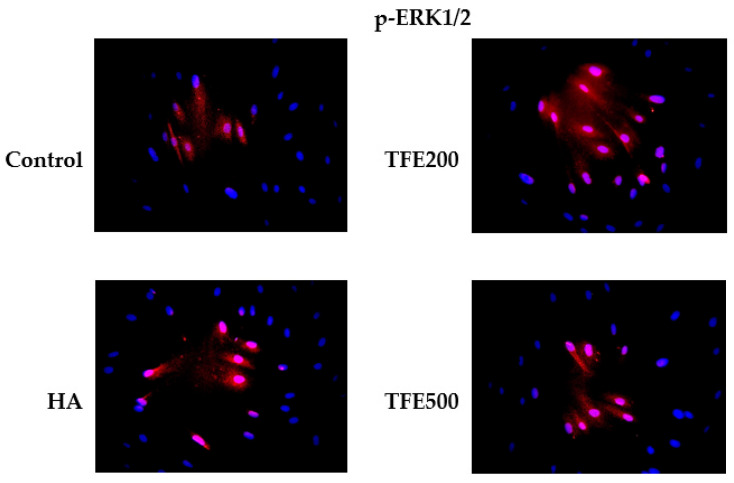
The phosphorylated Extracellular Signal-Regulated Kinases (p-ERK1/2) immunofluorescence in HDF treated with HA and TFE. Cells were treated with HA at 500 μg/mL or TFE at 200 and 500 μg/mL. The blue fluorescence channel shows cell nuclei, whereas the red fluorescence channel represents prolidase fluorescence. Images were acquired using a 20× objective. One microscopic field per condition was analyzed. Quantitative analysis of the corresponding original fluorescence images is presented in Table 6. For presentation purposes, identical brightness, contrast, and sharpening adjustments were applied uniformly to all displayed images; quantitative analysis was performed using the original unprocessed images.

To further characterise signalling-related cellular responses associated with scratch closure, phosphorylated ERK1/2 immunofluorescence was evaluated (Figure 10). Descriptive quantitative image analysis showed that background-corrected p-ERK1/2 fluorescence intensity per Hoechst-positive nucleus was 121.0% of the control value in the HA group, 124.6% in the TFE200 group and 134.9% in the TFE500 group. The normalised quantitative values are presented in Table 6, while the complete image analysis measurements, including analysed area, integrated density, mean background fluorescence, nuclei count and background-corrected integrated fluorescence, are provided in the Supplementary Materials (Table S6).

The TFE500 image showed the highest p-ERK1/2 fluorescence. This result was consistent with the higher 24 h scratch-closure value observed for TFE500. However, as the immunofluorescence analysis was based on a single image per condition, these findings should be considered descriptive rather than statistically confirmed changes in protein expression, definitive ERK1/2 pathway activation, or evidence of a causal relationship between p-ERK1/2 fluorescence and scratch closure.

## 3. Discussion

### 3.1. Effects of TFE and HA on HDF Viability, DNA Biosynthesis, and p-AKT/p-mTOR Immunofluorescence

The finding of this part of the study was that neither TFE nor HA produced an evident reduction in HDF viability, whereas TFE significantly increased DNA biosynthesis at both tested concentrations. In contrast, HA at 500 µg/mL had no significant effect on DNA biosynthesis. These results suggest that the tested preparations were well tolerated under the experimental conditions applied and that TFE, but not HA, induced a measurable response in DNA synthesis.

The MTT values remained close to those of the untreated control in all treatment groups. However, since the viability assay was carried out using five technical replicate wells within a single experiment, these results should be interpreted descriptively as showing no obvious treatment-related reduction in metabolic activity. Furthermore, the MTT assay reflects cellular reduction capacity rather than cell number alone, so it does not rule out more subtle alterations in cellular function. Nevertheless, the absence of an evident decrease in MTT activity supports the use of the selected TFE and HA concentrations in subsequent functional assays. The scratch assay does not allow discrimination between enhanced cell migration and increased proliferation; therefore, both processes may have contributed to wound closure.

TFE200 and TFE500 increased DNA biosynthesis by approximately 130% and 141%, respectively, whereas HA produced a value of around 107%. The incorporation of [methyl-^3^H]thymidine into DNA is commonly used as an indirect indicator of cell-cycle progression and proliferative activity. Therefore, the present findings suggest that TFE may promote a growth-related response in HDFs under the applied conditions. However, DNA synthesis alone does not demonstrate proportional increases in cell number.

The results are broadly consistent with previous studies describing the protective and growth-promoting effects of isolated TFP in fibroblasts. Shen et al. [17] demonstrated that an isolated TFP preparation reduced H_2_O_2_-induced oxidative stress and apoptosis in human skin fibroblasts, which was associated with increased SIRT1 expression and Akt-related signalling. In another study, *Tremella fuciformis* polysaccharides reduced UVA-induced damage in human dermal fibroblasts through responses involving the Nrf2/Keap1 pathway [18]. These findings provide a biological context for the continued viability and increased DNA synthesis observed following TFE treatment.

Nevertheless, direct comparison with those studies requires caution. Previous experiments have used isolated or more specifically characterised polysaccharide preparations, often in oxidative or ultraviolet injury models [25]. In contrast, the present study examined a commercially available, cosmetic-grade TFE under basal culture conditions. Although the tested preparation was described by the manufacturer as polysaccharide-rich, it was neither independently fractionated nor chemically characterised in detail. Therefore, the observed cellular effects should be attributed to the commercial TFE as a whole, rather than to TFP or the molecular mechanisms reported for isolated polysaccharide fractions.

The absence of a significant increase in DNA biosynthesis following HA treatment should not be interpreted as evidence of biological inactivity. HA acts as both a structural component of the extracellular matrix and a signalling molecule through receptors such as CD44 and RHAMM [21]. Its biological effects depend on molecular weight, concentration, exposure time, receptor availability, cell type, and extracellular matrix context [26]. The HA used in the present study had a molecular weight of 1.5–2.2 MDa and therefore represented a high-molecular-weight preparation.

HA molecules of different sizes may produce distinct fibroblast responses. Native HA and HA fragments have been shown to differ in their effects on fibroblast adhesion, proliferation, migration, and the expression of extracellular matrix-related genes [27]. High-molecular-weight hyaluronan is generally associated with structural, hydrating, pericellular matrix and homeostatic functions. In contrast, lower-molecular-weight fragments may induce different receptor-mediated proliferative, migratory or inflammatory responses [28]. The absence of a marked mitogenic response in the present experiment may therefore be due to the high molecular weight of the HA preparation, its concentration of 500 µg/mL, the exposure period of 24 h, or the receptor context of the HDF. HA may also influence extracellular matrix metabolism without producing a parallel increase in proliferation. Previous studies have shown that HA inhibits fibroblast proliferation while stimulating collagen and non-collagen protein synthesis, indicating that its effects on cell growth and matrix production may be independent of each other [28]. This observation is relevant to the present response profile, in which HA did not significantly increase DNA biosynthesis but significantly increased collagen biosynthesis, as discussed in Section 3.2. Under the applied conditions, HA may therefore have affected matrix-associated processes more strongly than fibroblast proliferation.

A descriptive quantitative analysis of the immunofluorescence images revealed higher p-AKT fluorescence in the TFE200 and TFE500 images compared to the control image. In contrast, p-mTOR fluorescence remained close to the control level. This was consistent with the increase in DNA biosynthesis observed following TFE treatment. This finding is also broadly consistent with a study by Shen et al., who reported increased AKT phosphorylation in human skin fibroblasts treated with an isolated fuciformis polysaccharide preparation in an H_2_O_2_-induced injury model [17].

However, the present immunofluorescence findings do not demonstrate the activation of the entire AKT/mTOR pathway. The quantitative image analysis was descriptive, based on one microscopic field per condition. Furthermore, no parallel increase in p-mTOR fluorescence was observed. Total AKT and total mTOR levels were not measured, nor were pathway-specific inhibition experiments performed. Fluorescence intensity differences may also be influenced by cell density, morphology, intracellular protein localisation, epitope accessibility and image capture conditions.

Accordingly, the higher p-AKT fluorescence should be interpreted as an associative observation rather than as evidence of statistically confirmed AKT activation or of AKT signalling mediating the increase in DNA biosynthesis. Confirmation would require independent biological replicates, Western blotting, or another quantitative protein assay; measurement of phosphorylated proteins relative to their corresponding total protein levels; and inhibition of AKT-related signalling. Therefore, the p-AKT/p-mTOR immunofluorescence data were used only to support the interpretation of the DNA-biosynthesis results and were not treated as a statistically validated mechanistic endpoint.

Overall, TFE significantly increased DNA biosynthesis and was associated with higher descriptive p-AKT fluorescence, whereas HA did not produce a comparable DNA synthesis response. These results support the possibility of a growth-related effect of TFE under the tested conditions but do not establish a direct molecular cause. The different response to HA may be related to its high molecular weight, which may have influenced extracellular matrix organisation and remodelling more than proliferation during the 24 h exposure period.

### 3.2. Effects of TFE and HA on Collagen Biosynthesis, Prolidase Activity, and β1-Integrin and IGF-1R Immunofluorescence

This part of the study found that HA and TFE500 significantly increased collagen biosynthesis, whereas TFE200 did not differ significantly from the control. Following HA treatment, collagen biosynthesis reached 141.2% of the control value, and following treatment with TFE500, it reached 135.6%. These results suggest that the higher concentrations of TFE tested were associated with increased collagen production under the experimental conditions applied, but not at a concentration of 200 μg/mL.

Collagen is the primary structural component of the dermal extracellular matrix, playing a crucial role in skin tensile strength, tissue integrity, and wound repair. Dermal fibroblasts are the principal cells responsible for collagen synthesis and extracellular matrix turnover; therefore, modulation of fibroblast collagen metabolism is relevant to tissue regeneration and the ageing process of the skin [29].

The response profile differed between TFE and HA. TFE was more strongly associated with increased DNA biosynthesis, whereas HA produced the highest numerical increase in collagen biosynthesis. This suggests that the preparations may affect different aspects of fibroblast function selectively. However, proliferation and extracellular matrix production are interconnected and context-dependent processes. The present results do not demonstrate that increased DNA biosynthesis directly promotes or limits collagen synthesis.

The collagen-stimulatory effect of HA is consistent with previous observations that HA can modulate fibroblast matrix metabolism independently of its effects on proliferation. Mast et al. [28] reported that hyaluronic acid (HA) inhibited fibroblast proliferation while increasing collagen and non-collagen protein synthesis. In vivo studies have also shown that cross-linked HA can stimulate the production of new dermal collagen, potentially by restoring mechanical support and allowing fibroblast stretching within the extracellular matrix [30]. These findings suggest that under the conditions used in the present study, high-molecular-weight HA may have a stronger effect on matrix-associated processes than on DNA biosynthesis.

However, the effect of TFE500 on collagen biosynthesis should be interpreted more cautiously, as the tested material was a commercial extract rather than an isolated or chemically defined polysaccharide fraction. Previous studies and reviews have associated *Tremella fuciformis*-derived preparations with antioxidant, photoprotective, wound-supportive and extracellular matrix-related effects [11,17,18]. However, the composition and biological activity of fungal extracts may vary according to source material, extraction, purification, molecular-weight distribution, and monosaccharide composition [11]. Because the commercial TFE was characterised mainly using manufacturer documentation, the increase in collagen biosynthesis should be attributed to the commercial TFE preparation as a whole and cannot be assigned exclusively to TFP or to specific bioactive constituents.

A descriptive immunofluorescence analysis showed higher levels of β1-integrin fluorescence in the HA and TFE500 images than in the control image. IGF-1R fluorescence was also higher in these images, with the highest value observed in the TFE500 image. While these patterns were consistent with the collagen-biosynthesis results, they did not demonstrate that either β1-integrin or IGF-1R mediated the collagen response.

β1-integrin contributes to cell–matrix adhesion and can transmit extracellular matrix-derived signals to pathways that regulate fibroblast survival, migration and collagen synthesis [22]. IGF-1R is also involved in anabolic signalling and the regulation of collagen production in fibroblasts [22,29]. Experimental studies have shown that alterations in collagen biosynthesis may occur alongside changes in β1-integrin and IGF-1R signalling. For instance, inhibiting collagen biosynthesis in human skin fibroblasts has been linked to decreased β1-integrin, IGF-1R and ERK1/2 expression. Conversely, other experimental conditions have been shown to increase collagen synthesis alongside IGF-1R-dependent signalling [31,32]. The present immunofluorescence analysis was based on one microscopic field per experimental condition. Differences in fluorescence intensity may reflect protein abundance, localisation, cell density, cell morphology, or image-acquisition factors. Furthermore, receptor activation was not measured directly. Therefore, the observed patterns should be interpreted as descriptive associations rather than statistically confirmed changes in receptor expression or evidence of receptor-mediated collagen regulation. Confirmation would require independent biological replicates, Western blotting, or another quantitative protein assay; assessment of receptor phosphorylation; and receptor- or pathway-specific inhibition experiments.

Prolidase contributes to collagen turnover by catalysing the final step in the degradation of imidodipeptides containing C-terminal proline or hydroxyproline. The released proline can be recycled for collagen resynthesis, linking prolidase activity to collagen metabolism and extracellular matrix renewal [5]. However, collagen production is also regulated at transcriptional, translational, post-translational and extracellular levels. Therefore, prolidase activity should not be regarded as a direct substitute for collagen biosynthesis.

In the present study, prolidase activity was 125% of the control value in the HA group and 110% in the TFE500 group, while TFE200 was 90%. Prolidase fluorescence showed a similar general pattern, with the highest value observed in the HA image, followed by the TFE500 image, and the lowest value observed in the control image. This agreement in trend with collagen biosynthesis suggests a possible association between increased collagen-related activity and prolidase-dependent proline recycling, particularly following HA and TFE500 treatment. Prolidase has also been linked to signalling involving EGFR, β1-integrin, IGF-1R, PI3K/Akt, and ERK1/2 [22]. Experimental modulation of prolidase activity in fibroblasts has been accompanied by corresponding changes in collagen biosynthesis and β1-integrin or IGF-1R signalling [22,32]. Extracellular prolidase has additionally been reported to induce anabolic responses and increase collagen biosynthesis in both control and experimentally wounded fibroblasts through signalling associated with EGFR [22].

However, the prolidase activity measurements in the present study were obtained from pooled samples and could not be subjected to inferential statistical analysis. The prolidase immunofluorescence analysis was also based on a single image per condition. Consequently, the apparent agreement between collagen biosynthesis, prolidase activity and prolidase fluorescence should be considered descriptive and suggestive of hypotheses. The data do not demonstrate that prolidase directly induces the collagen response to HA or TFE500.

Overall, HA and TFE500 significantly increased collagen biosynthesis, whereas TFE200 had no significant effect. HA exhibited the highest descriptive prolidase activity and fluorescence values, while TFE500 was associated with higher β1-integrin, IGF-1R and fluorescence than the control image. These findings suggest that HA and the higher TFE concentration may support collagen-related fibroblast activity through partially overlapping cellular responses. However, the available data do not establish a specific receptor-dependent or prolidase-mediated mechanism. Consequently, prolidase activity and immunofluorescence findings are interpreted as descriptive, whereas collagen biosynthesis represents the quantitatively validated endpoint in this part of the study.

### 3.3. Effects of TFE and HA on Scratch-Wound Closure and p-ERK1/2 Immunofluorescence

The scratch assay revealed that the TFE500 group exhibited the highest mean scratch-wound closure value at 24 h. Mean closure reached 39.70% following treatment with TFE500, compared with 27.33% for TFE200, 24.30% for hyaluronic acid (HA), and 18.66% for the untreated control group. However, these measurements were obtained from technical replicate wells within a single experiment. Therefore, the observed differences should be interpreted as descriptive trends rather than statistically confirmed treatment effects.

The in vitro scratch assay provides a simplified model for monitoring the reduction of a cell-free area in a confluent monolayer. Although it is commonly used to investigate cell migration, scratch closure may also be influenced by cell spreading, survival, changes in morphology and proliferation, particularly when proliferation is not pharmacologically inhibited [33]. Accordingly, the present results should be described as scratch-wound closure rather than fibroblast migration alone.

This distinction is particularly important when interpreting the TFE response. TFE200 and TFE500 were found to significantly increase DNA biosynthesis, indicating an increased DNA synthesis response under the applied conditions. As no proliferation inhibitor was used in the scratch assay, the higher mean closure values observed in the TFE-treated groups may partly result from increased proliferation, as well as possible changes in migration and spreading. However, the current experimental design does not allow us to determine the relative contributions of these processes.

Previous studies have shown that preparations derived from *Tremella fuciformis* may influence scratch closure in skin cell models. For example, Chiang et al. reported increased closure of scratch areas in human fibroblast and keratinocyte cultures treated with a *Tremella fuciformis* preparation [19]. This observation is consistent with the higher mean closure values for TFE observed in the present study. However, direct comparison requires caution due to differences in extract composition, treatment concentrations, cell models, exposure conditions and analytical procedures between studies. Furthermore, the commercial TFE used in this study was neither independently fractionated nor chemically characterised in detail; therefore, its effects on scratch closure should be attributed to the commercial TFE preparation as a whole and cannot be assigned exclusively to TFP.

The HA group also exhibited a higher mean closure value than the control group, albeit modest and not statistically significant. HA can influence fibroblast adhesion and motility through interactions with receptors such as CD44 and RHAMM. The strength of these responses depends on HA molecular weight, concentration, receptor availability and extracellular matrix context. Experimental evidence suggests that certain HA oligosaccharides can encourage dermal fibroblast migration and wound repair and that RHAMM-dependent signalling plays a role in fibroblast movement and organisation during tissue repair [24,34].

The HA used in the present study was a high-molecular-weight sodium hyaluronate preparation of 1.5–2.2 MDa. Its response cannot therefore be assumed to resemble that of low-molecular-weight HA or defined HA oligosaccharides. Under the present conditions, high-molecular-weight HA may have affected pericellular matrix organisation, adhesion, or cell spreading without producing a marked proliferative response. This interpretation is consistent with the absence of a significant increase in DNA biosynthesis and with the stronger effect of HA on collagen biosynthesis. Nevertheless, CD44, RHAMM, focal-adhesion signalling, and cytoskeletal organisation were not evaluated, and the mechanism underlying the HA-associated closure pattern remains uncertain.

Representative images taken at 48 and 72 h showed extensive or nearly complete narrowing of the scratch area in all groups. The remaining cell-free regions were too small and irregular to be reliably delineated in Fiji/ImageJ, so these later observations were evaluated qualitatively instead. They document the later course of scratch closure but do not permit quantitative comparison of treatment kinetics. Additional early and intermediate time points would be more informative because they would capture group differences before the scratch boundaries become difficult to identify.

A descriptive quantitative immunofluorescence analysis showed that the percentage of p-ERK1/2-associated fluorescence compared to the control value was 121.0% in the HA image, 124.6% in the TFE200 image and 134.9% in the TFE500 image. Therefore, the highest fluorescence value was observed for TFE500, which was also the group with the highest mean 24 h scratch-closure value. ERK1/2 signalling is involved in several processes relevant to scratch closure, including cell-cycle progression, survival, cytoskeletal reorganisation, and migration [35,36]. Experimental activation of ERK has been associated with increased movement of fibroblasts or keratinocytes, and inhibition of ERK signalling has reduced migration in selected cellular models [35].

The similarity in direction between the TFE500 scratch-closure and p-ERK1/2 fluorescence values suggests an association between ERK-related cellular responses and scratch closure. However, the present findings do not demonstrate activation of the ERK1/2 pathway or establish that the closure response is mediated by ERK1/2. Only one image was analysed per condition; total ERK1/2 was not measured; phospho-to-total ERK1/2 ratios were not calculated; and no MEK/ERK inhibitor was not used. Furthermore, since proliferation was not controlled, higher p-ERK1/2 fluorescence could be related to proliferation, migration, survival, or a combination of these processes.

The current assay also represents only one component of wound repair. Physiological wound healing involves coordinated inflammatory, proliferative, angiogenic and remodelling phases, with contributions from fibroblasts, keratinocytes, endothelial cells, immune cells and the extracellular matrix [3,6]. The present monoculture scratch assay did not assess inflammatory cytokines, oxidative stress, angiogenesis, matrix degradation, myofibroblast differentiation, or later remodelling. Therefore, the findings should not be generalised to the complete in vivo wound-healing process.

Overall, TFE500 exhibited the highest descriptive mean scratch-wound closure and p-ERK1/2 fluorescence values under the applied conditions. As TFE also increased DNA biosynthesis, enhanced proliferation may have contributed to the closure pattern, and the result cannot be attributed exclusively to migration. HA produced a more moderate closure response and may have influenced matrix- and adhesion-related fibroblast functions rather than proliferation. Further studies should include independent biological experiments, proliferation-controlled scratch assays, time-lapse cell tracking, additional early time points, Transwell migration assays and quantitative analysis of CD44/RHAMM, focal adhesion, cytoskeletal and ERK-related signalling. Thus, the scratch-wound and p-ERK1/2 data should be regarded as supportive observations rather than definitive evidence of a migration-specific or ERK1/2-dependent mechanism.

### 3.4. Limitations of the Study and Future Perspectives

The present study has several limitations. It was first conducted in vitro on a limited experimental scale. The commercial TFE was characterised primarily based on the manufacturer’s documentation, rather than being independently subjected to detailed chemical profiling, fractionation or molecular-weight analysis. Therefore, the composition of individual batches and the contribution of specific constituents could not be determined, so the observed effects should be attributed to the commercial TFE preparation as a whole and cannot be assigned exclusively to TFP or to specific bioactive constituents.

Furthermore, only two TFE concentrations and one HA concentration were evaluated. While the selected concentrations were well tolerated under the applied conditions, a broader dose–response analysis is required to determine the minimum effective concentration, the optimal concentration range and the shape of the concentration-response relationship.

Several analyses had limited replication. Immunofluorescence quantification was based on one image per experimental condition, prolidase activity was measured in pooled samples, and scratch-wound closure was evaluated using technical replicates within a single experiment. Quantitative scratch analysis was restricted to the 0–24 h interval because the remaining cell-free areas at 48 and 72 h could not be defined reliably. These findings should therefore be interpreted cautiously.

The study did not include complementary gene and protein expression analyses, phospho-to-total protein measurements or pathway-specific inhibition experiments. Markers related to extracellular matrix remodelling, angiogenesis, inflammation, and scar formation, including collagen III, hyaluronan synthases, prostaglandin E_2_, matrix metalloproteinases, tissue inhibitors of metalloproteinases, and inflammatory cytokines, were not assessed.

Finally, the experiments were performed under standard culture conditions and did not incorporate oxidative, inflammatory, ultraviolet or other injury-related models. The scratch assay reflects the combined effects of migration, spreading and proliferation in a fibroblast monoculture but does not reproduce the complete, multi-stage wound-healing process. Future studies should therefore include biological replication, a broader range of doses and times, detailed characterisation of extracts, oxidative and inflammatory models, additional remodelling-related endpoints and complementary mechanistic analyses.

## 4. Materials and Methods

### 4.1. Materials

#### 4.1.1. *Tremella fuciformis* Extract

A commercially available cosmetic-grade powdered extract of *Tremella fuciformis* fruiting bodies (INCI: *Tremella fuciformis* Sporocarp Extract; HyaCare^®^ Tremella, Evonik Operations GmbH, Essen, Germany; batch GS241122) was used as the test material. The extract was supplied by the manufacturer as a water-soluble white-to-ivory powder. The authors did not perform the extraction, purification, fractionation, or chemical standardisation of the material. The commercial product was used as received, without additional purification or fractionation before the experiments.

According to the manufacturer’s documentation, the product is obtained from the sporocarp of *Tremella fuciformis* by extraction and subsequent purification. The preparation is described as a polysaccharide-rich fungal-derived material containing at least 65% total saccharides, with sugars accounting for more than 80% of the declared carbohydrate fraction. The manufacturer reports an average molecular weight of approximately 1000 kDa and a glucuronic acid content of 13–30%. No preservatives or auxiliary additives are declared in the formulation.

The manufacturer’s specifications also include limits for total heavy metals (≤20 ppm), arsenic (≤2 ppm), residual ethanol (≤2%), and microbiological contamination (total viable count ≤200 CFU/g, with the absence of specified pathogens). According to the accompanying documentation, the product was manufactured without genetically modified organisms or animal-derived components.

Product identity and batch quality information were obtained from the manufacturer’s specifications and accompanying analytical documentation. The documentation reported identification as *Tremella fuciformis* by DNA barcoding with 98.2% sequence similarity and compliance with the declared chemical and microbiological quality criteria. These parameters were not independently verified by the authors. Therefore, although the commercial preparation was described by the manufacturer as polysaccharide-rich, no independent compositional or physicochemical characterisation was performed in the present study, and the observed biological effects should be attributed to the commercial TFE preparation as a whole rather than exclusively to TFP or to specific bioactive constituents.

The concentrations of TFE were selected based on previously published studies evaluating *Tremella fuciformis* polysaccharides or extracts in skin-related cellular models, together with preliminary cell-viability results confirming that the selected concentrations were non-cytotoxic under the present experimental conditions [17,18,25]. The preparation of the TFE treatment in medium is described in Section 4.2.2.

#### 4.1.2. Hyaluronic Acid

Sodium hyaluronate (CAS 9067-32-7; ≥94% on a dry basis; molecular weight 1.5–2.2 MDa) was used as the reference compound and was purchased from Pol-Aura (Acros Organics, Pol Aura, Morag, Poland; catalogue no. PA-07-25177). Hereafter, sodium hyaluronate is referred to as HA. HA was prepared in the same medium and used at a final concentration of 500 µg/mL. The preparation of the HA treatment medium is described in Section 4.2.2.

#### 4.1.3. List of Reagents

HyaCare^®^ Tremella (Evonik Operations GmbH, Essen, Germany; Product Code/Mat-No: 99138404), Sodium hyaluronate (Acros Organics, Pol Aura, Morag, Poland; catalogue no. PA-07-25177), DMEM, high glucose, pyruvate (Thermo Fisher/Gibco, Thermo Fisher Scientific, Waltham, MA, USA; catalogue no. 11995065); Foetal Bovine Serum (FBS) (Thermo Fisher/Gibco, Thermo Fisher Scientific, Waltham, MA, USA; catalogue no. A5256701), Penicillin-Streptomycin (10,000 U/mL) (Gibco, Waltham, MA, USA; catalogue no. 15140122), Thiazolyl Blue Tetrazolium Bromide (MTT) (Gibco, Waltham, MA, USA; catalogue no. M6494), PBS (Gibco, Waltham, MA, USA; catalogue no. M2128-100MG), Phosphate-buffered saline (PBS, 1X), sterile-filtered (Thermo Fisher Scientific, Waltham, MA, USA; catalogue no. J61196.AP), DMSO (Chempur, Piekary Slaskie, Poland; catalogue no. 5906076470745), [methyl-^3^H]thymidine (PerkinElmer, Waltham, MA, USA), methyl-^3^H] Thymidine 5mCi (Moravek Inc. Brea, CA, USA; catalogue no. MT6037), [2,3,4,5-^3^H]L-Proline 5mCi (Moravek Inc. Brea, CA, USA; catalogue no. MT522M), Collagenase from Clostridium histolyticum Type I, ≥125 CDU/mg (Sigma-Aldrich, St. Louis, MO, USA; catalogue no. C0130-100MG), Triton X-100 (Sigma-Aldrich, St. Louis, MO, USA; catalogue no. X100-5ML), Gly-Pro (Sigma-Aldrich, St. Louis, MO, USA; catalogue no. G3002-10G), Ultima Gold™ XR LSC Cocktail (Sigma-Aldrich, Saint Louis, MO, USA; catalogue no. L8411-2X5L), Hoechst 33342, trihydrochloride trihydrate solution (20 mM) (Life Technologies Pitampura, Delhi, India; catalogue no. 40047); Prolidase mouse antibody (BD Biosciences, Franklin Lakes, NJ, USA; catalogue no. 552859), p-ERK1/2 mouse antibody (BD Biosciences; catalogue no. 612358), β1-integrin mouse antibody (BD Biosciences; catalogue no. 610467), p-Akt mouse antibody (BD Biosciences; catalogue no. 612392), p-mTOR mouse antibody (BD Biosciences; catalogue no. 612624), and IGF-1R rabbit antibody (BD Biosciences; catalogue no. 9750)., Alexa Fluor 594-conjugated anti-mouse IgG and anti-rabbit IgG secondary antibodies (Invitrogen™, Thermo Fisher Scientific, Waltham, MA, USA; catalogue nos. A-11005 and A-11012).

### 4.2. Methods

#### 4.2.1. Cell Lines and Culture

Primary Dermal Fibroblasts; Normal, Human, Adult (HDFa) (ATCC^®^ catalogue no. PCS-201-012™) were purchased from the American Type Culture Collection (ATCC, Manassas, VA, USA). According to the supplier’s documentation, the cells were isolated from adult skin tissue (growth properties: Adherent Cells per vial: ≥ 5.0 × 10^5^). The cells were propagated in DMEM containing 10% foetal bovine serum, penicillin at a final concentration of 50 U/mL, and streptomycin at a final concentration of 50 µg/mL. Routine cultures were maintained in 100 mm culture dishes containing 10 mL of complete medium. Cells were incubated at 37 °C in a humidified atmosphere containing 5% CO_2_. The culture medium was renewed every 3 days. HDFs between passages 10 and 15 were used throughout the experiments. This passage range and the general culture conditions were selected on the basis of the authors’ previously published cell-culture procedure [29] and were applied consistently in the present study to reduce variability related to culture history. The previously described protocol was adapted to the current experimental design.

#### 4.2.2. TFE and HA Treatments

Fresh treatment preparations were made immediately before each experiment using DMEM as the vehicle. TFE was dissolved in DMEM to prepare a 1 mg/mL stock solution, which was gently mixed to avoid excessive foaming and then diluted in culture medium to obtain final concentrations of 200 and 500 µg/mL. HA was weighed and added directly to DMEM to obtain a final concentration of 500 µg/mL and was also mixed gently before application. The concentrations were selected based on preliminary experiments or published studies showing absence of cytotoxicity while allowing detection of biological responses.

For each experimental vessel, an appropriate volume of treatment medium was prepared so that, after addition to the corresponding well or culture dish, the final concentration of TFE or HA in the total culture-medium volume was 200, 500, or 500 µg/mL, respectively. The preparations were used immediately after preparation and were not stored. No additional filtration or sterilisation was performed. Control cells received the same volume of culture medium without TFE or HA.

#### 4.2.3. Cell Viability Assay

Cell viability was evaluated using the methylthiazolyl tetrazolium (MTT) assay, adapted from the method described by Carmichael et al. [37]. The assay is based on the reduction in the yellow tetrazolium salt MTT to insoluble purple formazan crystals by metabolically active cells. HDFs were seeded in 96-well plates and cultured until approximately 80% confluence was reached. The culture medium was then removed, and the cells were washed once with 1X PBS. Freshly prepared treatment medium containing TFE at 200 or 500 µg/mL or HA at 500 µg/mL was added to the appropriate wells. Control cells received the same volume of culture medium without TFE or HA. Five technical replicates were used for each condition within the same experiment. After 24h of incubation, the treatment medium was removed, and MTT solution was added to each well to obtain a final concentration of 5 mg/mL. The cells were incubated with MTT at 37 °C for 30 min. The MTT-containing medium was then removed, and the resulting formazan crystals were dissolved in DMSO 100 µL per well. The plate was gently mixed to ensure complete dissolution of the crystals. Absorbance was measured at 570 nm using a microplate reader. Cell viability was expressed as a percentage of the untreated control group based on the absorbance values. Data are presented as mean ± SD of five parallel wells (*n* = 5).

#### 4.2.4. DNA Biosynthesis Assay

DNA biosynthesis was evaluated by measuring the incorporation of [methyl-^3^H]thymidine into newly synthesised DNA, using a procedure adapted from the authors’ previously published methodology [38]. HDFs were seeded in 6-well plates at an initial density of 3 × 10^5^ cells per well and cultured until confluence. The culture medium was then removed, and the cells were washed once with 1X PBS. Fresh treatment medium containing TFE at 200 or 500 µg/mL or HA at 500 µg/mL was added to the appropriate wells, as described in Section 4.2.2. Control cells received the same volume of complete culture medium without TFE or HA. Immediately after the addition of the treatment medium, [methyl-^3^H]thymidine was added to each well at a final activity of 0.5 µCi/mL. The cells were incubated for 24 h at 37 °C in a humidified atmosphere containing 5% CO_2_. After incubation, the culture medium was removed, and the cells were washed twice with cold 1X PBS to eliminate unincorporated radiolabelled thymidine. The cells were then collected and processed for radiometric analysis. Radioactivity incorporated into DNA was measured using a Tri-Carb 2810 TR liquid scintillation analyser (PerkinElmer, Waltham, MA, USA) and expressed as disintegrations per minute (dpm). Three independent experiments were performed. In each experiment, three technical replicate wells were analysed per condition. The mean of the repeated measurements was calculated for each well, followed by calculation of the experiment-level mean. DNA biosynthesis was expressed relative to the untreated control, which was set to 100%.

#### 4.2.5. Biosynthesis of Collagen with 5-[^3^H]-Proline Incorporation

Collagen biosynthesis was evaluated by measuring the incorporation of 5-[^3^H]-proline into collagenase-sensitive proteins using a procedure adapted from Peterkofsky et al. [39] and the authors’ previously published methodology [29]. HDFs were seeded in 6-well plates at an initial density of 3 × 10^5^ cells per well and cultured until confluence. The cells were then treated with TFE or HA as described in Section 4.2.2. During the 24 h treatment period, 5-[^3^H]-proline was added to the culture medium at a final activity of 5 μCi/mL. After incubation, the cells were washed with 1xPBS at pH 7.4, collected in PBS supplemented with 10 mM non-radioactive proline, and stored at −80 °C until further analysis. Collagen digestion was performed using purified collagenase from Clostridium histolyticum. Radioactivity in the collagenase-sensitive and collagenase-resistant protein fractions was measured using a Tri-Carb 2810 TR liquid scintillation analyser (PerkinElmer, Waltham, MA, USA). Collagen biosynthesis was calculated from the collagenase-sensitive protein fraction, normalised to total protein synthesis, and expressed relative to the untreated control, which was set to 100%. Three independent experiments were performed. Data are presented as mean ± standard deviation (SD) and were subjected to statistical analysis as described in Section 4.2.11.

#### 4.2.6. Prolidase Activity Using Gly-Pro Substrate

Prolidase activity was determined using glycyl-L-proline (Gly-Pro) as the synthetic substrate, according to the procedure described by Myara et al. [40], with modifications appropriate to the present experimental design. HDFs were seeded in 100 mm culture dishes at a density of 1 × 10^6^ cells per dish and maintained until confluence. The cells were then exposed to TFE or HA as described in Section 4.2.2. After 24 h of treatment, the cells were collected and processed for determination of prolidase activity. The assay quantified the amount of proline released from Gly-Pro by the cellular enzyme preparation. Total protein concentration in the cell homogenate supernatants was measured using the Lowry method [41]. Prolidase activity was calculated as nanomoles of proline released per minute and normalised to the protein content of the corresponding sample, expressed in milligrams. For each experimental condition, material obtained from two culture dishes was pooled before the enzymatic assay. Consequently, replicate-level measurements were not available, and the prolidase activity results were evaluated descriptively without inferential statistical analysis.

#### 4.2.7. Immunofluorescence Staining and Confocal Microscopy

The immunofluorescence procedure was adapted from the authors’ previously published protocol [29] and modified for the experimental conditions of the present study. HDFs were cultured in black 96-well imaging plates until approximately 80% confluence was reached and were subsequently treated with TFE or HA as described in Section 4.2.2. After 24 h, the treatment medium was removed, and the cells were fixed with 3.7% formaldehyde for 10 min at room temperature. Following fixation, the wells were washed once with 1X PBS (100 µL per well). The cells were permeabilised with 0.1% Triton X-100 for 10 min and then washed twice with PBS. Nonspecific binding sites were blocked with 3% FBS for 30 min at room temperature. After removal of the blocking solution, 50 µL of the appropriate primary antibody diluted 1:1000 in 3% FBS was added to each well. The plate was incubated for 1 h at room temperature and subsequently washed three times with PBS. Alexa Fluor 594-conjugated secondary antibodies directed against the species of the corresponding primary antibodies were then added at a dilution of 1:1000 in a volume of 50 µL per well. The plate was incubated for 24 h at room temperature in the dark and washed three times with PBS. Cell nuclei were counterstained with Hoechst 33342 at a final concentration of 2 µg/mL in PBS. Images were acquired using a BD Pathway 855 confocal laser-scanning imaging system (Becton Dickinson, Franklin Lakes, NJ, USA) equipped with AttoVision 1.6 software and a 20× objective. Hoechst 33342 fluorescence was recorded in the blue channel, whereas Alexa Fluor 594-associated fluorescence was recorded in the red channel using the corresponding filter sets. Exposure time, illumination intensity, objective magnification, and other image-acquisition parameters were kept constant across all experimental groups analysed for the same marker. Original, unprocessed images were used for quantitative fluorescence analysis. Any adjustments applied to the displayed figures were used exclusively to improve visual presentation. Immunofluorescence experiments were exploratory and performed using one representative microscopic field from a single experiment per condition.

#### 4.2.8. Antibodies

The following primary antibodies were used for immunofluorescence analysis: prolidase mouse antibody, p-ERK1/2 mouse antibody, β1-integrin mouse antibody, p-Akt mouse antibody, p-mTOR mouse antibody, and IGF-1R rabbit antibody. The primary antibodies were used at a dilution of 1:1000.

Alexa Fluor 594-conjugated anti-mouse IgG and anti-rabbit IgG secondary antibodies were used at a dilution of 1:1000.

#### 4.2.9. Quantitative Analysis of Immunofluorescence Images

Immunofluorescence images were quantitatively analysed using Fiji/ImageJ 2.17.0 (license: GPLv3+; https://imagej.net/software/fiji/; accessed on 10 June 2026). According to described fluorescence-quantification procedures [42,43]. Corresponding Alexa Fluor 594 and Hoechst 33342 channel images representing the same microscopic field were used for each condition. Original, unprocessed images were inspected for artefacts, saturation, uneven illumination, and dimensional consistency. When required, RGB images were separated using the Split Channels function. The red channel was used to quantify protein-associated fluorescence, whereas the blue channel was used to count Hoechst-positive nuclei. For nuclear counting, the blue channel was converted to 8-bit greyscale and segmented using Otsu thresholding. Binary masks were refined using the Fill Holes and Close functions when necessary, and Hoechst-positive nuclei were counted using Analyze Particles. Segmentation results were visually inspected to confirm that detected objects corresponded approximately to visible nuclei. Protein-associated fluorescence was measured in the entire red-channel image without thresholding. Area and integrated density were recorded. Background fluorescence was determined from three to five cell-free regions selected within each image, and the mean background intensity was calculated. Background-corrected integrated fluorescence was calculated as:


Corrected Total Cell Fluorescence= Integrated density − (analyzed area × mean background fluorescence)

The corrected fluorescence was normalised to the number of Hoechst-positive nuclei:


$$Corrected\;fluorescence\;per\;nucleus\;=\;\frac{corrected\;fluorescence}{number\;of\;Hoechst\;positive\;nuclei.}$$

For presentation, the control value was set to 100%, and treated conditions were expressed relative to the corresponding control:


$$Relative\;fluorescence\left(\%\right)=(\frac{corrected\;fluorescence\;per\;nucleus\;in\;the\;treated\;image\;}{\;corrected\;fluorescence\;per\;nucleus\;in\;the\;control\;image\;})\times \;100$$

Because only one image was available per condition, the analysis was descriptive. No error bars, *p*-values, or inferential statistical tests were applied. The results were interpreted as differences in marker-associated fluorescence intensity and not as statistically confirmed changes in protein expression or definitive evidence of pathway activation.

#### 4.2.10. Scratch-Wound Closure Assay

The scratch-wound closure assay was adapted to the experimental conditions of the present study based on previously published protocols and the authors’ earlier studies [29,44,45]. Confluent HDFs cultured in 6-well plates were subjected to a linear scratch using a sterile 200 µL pipette tip. To standardise scratch placement and direction, a single scratch was created through the centre of each well in one continuous motion using the same type of pipette tip. The edge of the plate lid was used as a straight guide to maintain a consistent central position and direction of the scratch across the wells.

After scratch formation, detached cells and cellular debris were removed by gently washing the wells with PBS. Fresh culture medium containing HA or TFE was then added as described in Section 4.2.2.

Microscopic images were acquired immediately after scratch formation (0 h) and after 24, 48, and 72 h using an inverted light microscope (Nikon, Minato, Tokyo, Japan) at 40× magnification. One microscopic field per well was recorded at each time point. The same central region of the scratch was followed over time whenever possible.

No pharmacological inhibitor of cell proliferation, such as mitomycin C, was used during the assay. Therefore, the reduction in scratch area may reflect the combined effects of cell migration, spreading, and proliferation. Accordingly, the measured endpoint is referred to as scratch-wound closure rather than fibroblast migration alone.

Wound closure was quantified using Fiji/ImageJ 2.17.0 (GPLv3+). For each field of view, a fixed region of interest (ROI) covering the central wound area was defined and applied identically to images acquired at 0 and 24 h. Images were converted to 8-bit greyscale and smoothed using a median filter (radius = 1). The wound area was segmented by thresholding (Otsu method) to isolate the cell-free region. Binary masks were refined using morphological operations (Fill Holes and Close). The wound area was measured using the Analyze Particles function with a size filter (Size = 100,000–∞ pixels^2^) to exclude small segmentation artefacts.

At 48 and 72 h, the remaining scratch region was extensively or nearly completely closed and became too small and irregular to be reliably detected and delineated using Fiji/ImageJ. Quantitative measurements at these later time points would therefore have introduced substantial uncertainty. Consequently, the 48 h and 72 h images were evaluated qualitatively and were not included in the quantitative analysis.

Wound area values were expressed in relative units (pixels^2^) and normalised to the initial wound area at 0 h. The percentage of wound closure (v%/24) and the wound closure rate (v%/h) were calculated as follows:

$$v\%/24=\frac{A0\;-A24}{A0}\;\times \;100\%$$

$$v\%/h\;=\;\frac{v\%/24}{24}$$

where A0—wound area at 0 h, A24—wound area at 24 h.

Images included in the figures were sharpened for presentation purposes only. All quantitative measurements were performed using the original, unprocessed image files.

#### 4.2.11. Statistical Analysis

Data from the DNA biosynthesis and collagen biosynthesis assays were obtained from three independent experiments and are presented as mean ± standard deviation (SD). Differences among the control, HA, TFE200, and TFE500 groups were analysed using one-way analysis of variance (ANOVA), followed by Dunnett’s multiple-comparisons test to compare each treatment group with the control. Adjusted *p*-values < 0.05 were considered statistically significant. MTT cell-viability data were obtained from five technical replicate wells within a single experiment and are presented descriptively as mean ± SD. Scratch-wound closure data were also obtained from technical replicate wells within a single experiment and are presented descriptively as mean ± SD. Immunofluorescence analysis was based on one image per condition, whereas prolidase activity was measured in pooled samples. These datasets were analysed descriptively and were not subjected to inferential statistical testing. The number and type of replicates are specified in the corresponding figure and table legends.

## 5. Conclusions

TFE and HA were well tolerated by HDFs under the applied experimental conditions and did not cause a significant reduction in cell viability. TFE significantly increased DNA biosynthesis at both concentrations that were tested, whereas HA did not have a significant effect on this parameter. In contrast, HA and TFE at 500 µg/mL significantly increased collagen biosynthesis, while TFE at 200 µg/mL did not differ significantly from the control. Descriptive immunofluorescence analysis showed elevated p-AKT fluorescence in the images subjected to TFE treatment, while p-mTOR fluorescence remained near the control level. Furthermore, elevated levels of β1-integrin, IGF-1R, prolidase, and p-ERK1/2 fluorescence were detected in specific treatment groups. However, because these analyses were based on a single image per condition, they should be regarded as associative and hypothesis-generating rather than as evidence of pathway activation or causal mechanisms. The mean scratch-wound closure value was observed to be highest for TFE500, followed by TFE200 and HA. The descriptive results of the scratch assay, which was based on technical replicates within a single experiment, do not establish a statistically confirmed treatment effect. Furthermore, scratch-wound closure may reflect of a combination of migration, spreading, and proliferation. The findings indicate that TFE and HA produced partially different but overlapping response profiles in HDF. TFE was found to be more strongly associated with increased DNA biosynthesis, whereas HA and TFE500 enhanced collagen biosynthesis. These results suggest that the commercial TFE preparation may support fibroblast functions relevant to dermocosmetic applications; however, further studies with independent biological replication, detailed extract characterisation, quantitative protein analyses, and mechanistic inhibition experiments are required to provide a more robust evidence base.

## Figures and Tables

**Table 1:** The dpm values of radioactive [methyl-3H] thymidine incorporation into DNA for *Tremella fuciformis* extract (TFE) and hyaluronic acid (HA). Human dermal fibroblasts (HDF) were treated with HA at 500 µg/mL and with TFE at concentrations of 200 and 500 µg/mL vs. the control. Three independent experiments were performed, with three technical replicates per condition in each experiment. For statistical analysis, the mean of the three technical replicates was calculated for each independent experiment. Data are presented as individual dpm measurements, experiment-level means, overall mean ± SD, and percentage of the control. Differences among groups were analysed using one-way ANOVA followed by Dunnett’s multiple-comparisons test, comparing each treatment group with the control. *p* < 0.05 was considered statistically significant (*).

Sample	Exp. 1: 1st	2nd	3rd	Mean	Exp. 2 1st	2nd	3rd	Mean	Exp. 3 1st	2nd	3rd	Mean	Overall Mean	SD	%	*p*-Value
Control	4139	5196	5118	4817.66	4119	4187	4103	4136.33	4127	4158	4142	4142.33	4365.44	391.64	100	-
HA	4081	4044	4070	4065	4994	4279	5236	4836.33	5220	5016	5218	5151.33	4684.22	558.91	107.3	0.639
TFE200	5916	5999	5907	5940.66	5820	5868	5814	5834	5252	5272	5262	5262	5678.88	364.95	130.08	0.0082 *
TFE500	6197	6198	6192	6195.67	6167	6162	6084	6137.66	6152	6158	6135	6148.33	6160.55	30.87	141.12	0.0013 *

**Table 2:** Descriptive quantitative analysis of phosphorylated protein kinase B (p-AKT) and phosphorylated mechanistic target of rapamycin (p-mTOR) immunofluorescence. Background-corrected integrated fluorescence was calculated as integrated density − (analysed area × mean background fluorescence). The corrected fluorescence was normalised to the number of Hoechst-positive nuclei and expressed relative to the control, which was set to 100%. One image per experimental condition was analysed; therefore, the results are descriptive, and no statistical analysis was performed.

p-AKT			p-mTOR		
Group	Corrected Fluorescence per Hoechst-Positive Nucleus	Relative Fluorescence (% of Control)	Group	Corrected Fluorescence per Hoechst-Positive Nucleus	Relative Fluorescence (% of Control)
Control	8.919	100.0	Control	19.30	100
HA	10.066	112.9	HA	15.05	78
TFE200	13.758	154.2	TFE200	19.20	99.5
TFE500	19.958	223.8	TFE500	19.12	99

**Table 3:** Collagen biosynthesis measurements for TFE and HA. Cells were treated with HA at 500 µg/mL or TFE at 200 and 500 µg/mL. Three independent experiments were performed. In each experiment, collagen biosynthesis was calculated as the difference (Δ) between the first and second measurement (Δ = 1st − 2nd) for the corresponding sample (**Δ^1^**, **Δ^2^**, and **Δ^3^** indicate experiments 1–3). Data are presented as individual measurements, experiment-level differences, overall mean ± standard deviation (SD), percentage of the untreated control, and *p*-values. Differences among groups were analysed using one-way ANOVA followed by Dunnett’s multiple-comparisons test, comparing each treatment group with the control. *p* < 0.05 was considered statistically significant (*).

Sample	1st	2nd	Δ^1^	1st	2nd	Δ^2^	1st	2nd	Δ^3^	Wpisz tutaj równanie. Overall Mean (Δ^1^ + Δ^2^ + Δ^3^)/3	SD	%	*p*-Value
Control	4006	1602	2404	4531	1752	2779	5525	2583	2942	2708.33	275.87	100	-
HA	5817	1972	3845	5765	1878	3887	5716	1974	3742	3824.66	74.60	141.22	0.00012 *
TFE200	4472	1688	2784	4589	1688	2901	3887	1120	2767	2817.33	72.95	104.02	0.767
TFE500	5605	1907	3698	5465	1957	3508	5740	1925	3815	3673.67	154.94	135.64	0.00021 *

**Table 4:** Descriptive quantitative analysis of β1-integrin and Insulin-Like Growth Factor-I Receptor (IGF-1R) immunofluorescence. Background-corrected integrated fluorescence was calculated as integrated density − (analysed area × mean background fluorescence). The corrected fluorescence was normalised to the number of Hoechst-positive nuclei and expressed relative to the control, which was set to 100%. One image per experimental condition was analysed; therefore, the results are descriptive, and no inferential statistical analysis was performed.

β1-Integrin			IGF-1R		
Group	Corrected Fluorescence per Hoechst-Positive Nucleus	Relative Fluorescence (% of Control)	Group	Corrected Fluorescence per Hoechst-Positive Nucleus	Relative Fluorescence (% of Control)
Control	31.52	100	Control	29.32	100
HA	44.73	141.96	HA	37	126.19
TFE200	39.78	126.22	TFE200	32.04	109.28
TFE500	46.28	146.85	TFE500	47.32	161.42

**Table 5:** Descriptive quantitative analysis of prolidase immunofluorescence. Background-corrected integrated fluorescence was calculated as integrated density − (analysed area × mean background fluorescence). The corrected fluorescence was normalised to the number of Hoechst-positive nuclei and expressed relative to the control, which was set to 100%. One image per experimental condition was analysed; therefore, the results are descriptive, and no inferential statistical analysis was performed.

Prolidase		
Group	Corrected Fluorescence per Hoechst-Positive Nucleus	Relative Fluorescence (% of Control)
Control	15.48	100
HA	29.33	189.47
TFE200	11.87	76.72
TFE500	24.15	156.04

**Table 6:** Descriptive quantitative analysis of phosphorylated Extracellular Signal-Regulated Kinases (ERK1/2) immunofluorescence. Background-corrected integrated fluorescence was calculated as integrated density − (analysed area × mean background fluorescence). The corrected fluorescence was normalised to the number of Hoechst-positive nuclei and expressed relative to the control, which was set to 100%. One image per experimental condition was analysed; therefore, the results are descriptive and no inferential statistical analysis was performed.

p-ERK1/2		
Group	Corrected Fluorescence per Hoechst-Positive Nucleus	Relative Fluorescence (% of Control)
Control	12.75	100
HA	15.42	120.95
TFE200	15.89	124.62
TFE500	17.19	134.85

## Data Availability

The data presented in this study are available in the main text of this article or on request from the corresponding author.

## Supplementary Materials

The following supporting information can be downloaded at: https://www.mdpi.com/article/10.3390/molecules31132354/s1, Table S1: The MTT test for TFE and HA. Cells were treated with HA at 500 µg/mL and with TFE at concentrations of 200 and 500 µg/mL vs. the control. Five technical replicates (five wells) were analyzed per condition. Data are presented as individual absorbance measurements, mean ± standard deviation (SD), and percentage of the control value; Table S2: Quantitative analysis of p-AKT and p-mTOR immunofluorescence in HDF treated with HA and TFE. The control value was set to 100%, and treated conditions were expressed relative to the corresponding control; Table S3: Quantitative analysis of β1- integrin and IGF-1R immunofluorescence in HDF treated with HA and TFE. The control value was set to 100%, and treated conditions were expressed relative to the corresponding control; Table S4: Quantitative analysis of prolidase immunofluorescence in HDF treated with HA and TFE. The control value was set to 100%, and treated conditions were expressed relative to the corresponding control; Table S5: Wound closure measurements in HDF cells during the first 24 h after scratching. Wound closure after 24 h expressed as % of the initial wound area. Wound closure rate expressed as %v/h. Cells were treated with HA at 500 µg/mL and TFE at 200 and 500 µg/mL. x^−^—mean. A0—wound area at 0 h. A24—wound area at 24 h. v%/24—the percentage of wound closure. v%/h—wound closure rate; Table S6: Quantitative analysis of p-ERK1/2 immunofluorescence in HDF treated with HA and TFE. The control value was set to 100%, and treated conditions were expressed relative to the corresponding control.

## Abbreviations

The following abbreviations are used in this manuscript:

ERK	Extracellular Signal-Regulated Kinases
Akt/p-AKT	Protein Kinase B/Phosphorylated Protein Kinase B
CD44/RHAMM	Receptor For Hyaluronan Mediated Motility
ECM	Extracellular Matrix
EGFR	Epidermal Growth Factor Receptor
FAK	Focal Adhesion Kinase
H_2_O_2_	Hydrogen peroxide
HA	Hyaluronic acid
HDF	Human Dermal Fibroblasts
IGF-1R	Insulin-Like Growth Factor 1 Receptor
MMP	Matrix Metalloproteinase
mTOR/p-mTOR	mechanistic target of rapamycin/phosphorylated mechanistic target of rapamycin
PI3K	Phosphoinositide 3-kinases
SIRT1	NAD-Dependent Deacetylase Sirtuin-1
TEWL	Transepidermal Water Loss
TFE	*Tremella fuciformis* Extract
TFP	*Tremella fuciformis* Polysaccharides
TIMP	Tissue Inhibitors of Metalloproteinases
UV	Ultraviolet Radiation
β1-integrin	Beta 1 Integrin
