# Supplementary material for: Tremella fuciformis Extract Evokes Similar Effect as Hyaluronic Acid on Wound Healing but Through Different Mechanisms in Human Dermal Fibroblasts
Source: Molecules. 2026 Jul 3;31(13):2354. doi: 10.3390/molecules31132354 (PMC13362964; doi:10.3390/molecules31132354)
Supplement: Supplementary file 1 [file molecules-31-02354-s001.zip › molecules-4363665-supplementary.pdf]

Table S1. The MTT test for TFE and HA. Cells were treated with HA at 500 μg/mL and with TFE at concentrations of 200 and 500 μg/mL vs. the control. Five technical replicates (five wells) were analyzed per condition. Data are presented as individual absorbance measurements, mean ± standard deviation (SD), and percentage of the control value.

Sample	1st	2nd	3rd	4th	5th	$\bar{x}$	% per cent	SD
Control	0.115	0.105	0.09	0.099	0.092	0.1002	100	0.0102
HA	0.093	0.093	0.094	0.094	0.106	0.096	95.80	0.0056
TFE200	0.085	0.091	0.084	0.118	0.103	0.0962	96.00	0.0143
TFE500	0.063	0.09	0.097	0.089	0.151	0.098	97.80	0.0323

Table S2. Quantitative analysis of p-AKT and p-mTOR immunofluorescence in HDF treated with HA and TFE. The control value was set to 100%, and treated conditions were expressed relative to the corresponding control.

p-AKT	Analysed Area	Integrated Density	Mean Background Fluorescence	Nuclei Count	Corrected Total Cell Fluorescence	Relative Fluorescence Per Nucleus	%
Control	244.668	267.143	0.014	26	231.913	8.919	100
HA	244.668	347.128	0.061	33	332.208	10.067	112.87
TFE200	244.668	526.906	0.073	37	509.15	13.760	154.28
TFE500	244.668	882.070	0.016	44	230.498	19.95	223.67

p-mTOR	Analysed Area	Integrated Density	Mean Background Fluorescence	Nuclei Count	Corrected Total Cell Fluorescence	Relative Fluorescence Per Nucleus	%
Control	244.668	668.095	0.048	34	656.35	19.30	100
HA	244.668	713.646	0.024	47	707.76	15.05	78
TFE200	244.668	770.847	0.010	40	768.39	19.20	99.5
TFE500	244.668	726.079	0.076	37	707.48	19.12	99

Table S3. Quantitative analysis of β1- integrin and IGF-1R immunofluorescence in HDF treated with HA and TFE. The control value was set to 100%, and treated conditions were expressed relative to the corresponding control.

β1-integrin	Analysed Area	Integrated Density	Mean Background Fluorescence	Nuclei Count	Corrected Total Cell Fluorescence	Relative Fluorescence Per Nucleus	%
Control	244.668	1050.268	0.041	33	1040.23	31.52	100
HA	244.668	996.34	0.050	22	984.106	44.73	141.96
TFE200	244.668	921.46	0.026	23	915.099	39.78	126.22
TFE500	244.668	973.282	0.005	21	972.062	46.28	146.85

IGF-1R	Analysed Area	Integrated Density	Mean Background Fluorescence	Nuclei Count	Corrected Total Cell Fluorescence	Relative Fluorescence Per Nucleus	%
Control	244.668	1028.78	0.023	35	1023.15	29.32	100
HA	244.668	893.478	0.022	24	888.098	37	126.19
TFE200	244.668	1064.74	0.030	33	1057.4	32.04	109.28
TFE500	244.668	1093.47	0.020	23	1088.58	47.32	161.42

Table S4. Quantitative analysis of prolidase immunofluorescence in HDF treated with HA and TFE. The control value was set to 100%, and treated conditions were expressed relative to the corresponding control.

Prolidase	Analysed Area	Integrated Density	Mean Background Fluorescence	Nuclei Count	Corrected Total Cell Fluorescence	Relative Fluorescence Per Nucleus	%
Control	244.668	559,73	0.010	36	557.29	15.48	100
HA	244.668	1158.48	0.032	39	1150.65	29.33	189.47
TFE200	244.668	303.87	0.077	24	285.03	11.87	76
TFE500	244.668	683.32	0.031	28	676.22	24.15	156.04

Table S5. Wound closure measurements in HDF cells during the first 24 h after scratching. Wound closure after 24 h expressed as % of the initial wound area. Wound closure rate expressed as %v/h. Cells were treated with HA at 500 µg/mL and TFE at 200 and 500 µg/mL. $\bar{x}$ - mean. A0 - wound area at 0 h. A24 - wound area at 24 h. v%/24 - the percentage of wound closure. **v%/h** -wound closure rate.

**Sample**	**1**					**2**					**3**						
Measurements	**A0**	**A24**	$^{1st}\bar{x}$	**v%/24**	**v%**	**A0**	**A24**	$^{2nd}\bar{x}$	**v%/24**	**v%**	**A0**	**A24**	$^{3rd}\bar{x}$	**v%/24**	**v%**	**$\bar{x}$v% ($^{1st}\bar{x}$+$^{2nd}\bar{x}$ + $^{3rd}\bar{x}$)/3**	**v%/24h**
Control	5521857	4072480	1449377	26.24	1.09	5754381	5198351	556030	9.66	0.40	5877747	4698203	1179544	20.06	0.83	0.777	18.659
HA	6725821	4763483	1962338	29.17	1.21	6622206	5262882	1359324	20.52	0.85	6471566	4970217	1501349	23.19	0.96	1.012	24.3
TFE200	5913893	3796701	2117192	35.81	1.49	6213170	4221901	1991269	32.04	1.33	5486151	4709826	776325	14.150	0.58	1.138	27.333
TFE500	6269777	2700956	3568821	56.92	2.37	6099314	3805348	2293966	37.61	1.56	6103337	4603074	1500263	24.58	1.024	1.654	39.704

Table S6. Quantitative analysis of p-ERK1/2 immunofluorescence in HDF treated with HA and TFE. The control value was set to 100%, and treated conditions were expressed relative to the corresponding control.

**p-ERK1/2**	**Analysed Area**	**Integrated Density**	**Mean Background Fluorescence**	**Nuclei Count**	**Corrected Total Cell Fluorescence**	**Relative Fluorescence Per Nucleus**	**%**
Control	244.668	412.27	0.069	31	395.39	12.75	100
HA	244.668	501.09	0.031	32	493.50	15.42	120.95
TFE200	244.668	532.13	0.031	33	524.55	15.89	124.62
TFE500	244.668	559	0.036	32	550.20	17.19	134.85
